# Evaluating initial screening practices for calcium dysregulation after acute traumatic spinal cord injury: a retrospective review

**DOI:** 10.1038/s41394-024-00663-0

**Published:** 2024-07-31

**Authors:** Rajbir Chaggar, Ranjodh Gill

**Affiliations:** 1 Department of Physical Medicine and Rehabilitation, VCU Health, Richmond, VA USA; 2 Department of Internal Medicine, Division of Endocrinology, VCU Health, Central Virginia Veteran’s Affairs Healthcare System, Richmond, VA USA

**Keywords:** Rehabilitation, Calcium and vitamin D

## Abstract

**Objectives:**

The purpose of this study was to determine the frequency of which calcium homeostasis markers are obtained in the acute setting after an initial traumatic spinal cord injury (TSCI).

**Design:**

Retrospective chart review of a limited data set linking ICD 10 codes designating TSCI to corresponding calcium homeostasis markers for patients with an initial chart encounter for TSCI.

**Setting:**

A level 1 trauma center in Virginia, United States

**Methods:**

The statistical software SPSS was used to calculate summary statistics including frequency, mean, and standard deviation for calcium homeostasis markers (basic metabolic panel, magnesium, spot urine calcium, testosterone panel, liver function tests, Vitamin D level, C-telopeptide, parathyroid hormone, celiac panel, DXA imaging report) as well as the mean and standard deviation for time to first check of the marker.

**Results:**

Most markers were not obtained besides calcium. Only 10 of 80 (12.5%) of subjects had a Vitamin D level (mean 28, SD 23) checked during acute admission (mean days to check 1.5, SD 1.6), with most other markers checked much less frequently.

**Conclusions:**

Most calcium homeostasis markers were not checked on acute admission after TSCI. Future studies on implementing a standardized calcium homeostasis marker protocol for monitoring and potential medical intervention should be explored.

## Introduction

Patients with traumatic spinal cord injuries and disorders (SCI/D) are known to suffer multisystem sequelae after initial traumatic neurologic event. Of particular interest is bone health and calcium homeostasis after SCI/D, particularly Vitamin D deficiency. It is known that those with SCI/D are at a higher risk of bone loss and fracture thought to be related to neuromuscular involvement, reduced mobility, and injury during transfer [1, 2]. It has been theorized that bone loss in acute traumatic SCI/D, coupled with hypercalcemia from immobilization, is related to a loss of balanced bone remodeling and increased level of bone resorption, with peak bone resorption occurring between 1 to 4 months post-acute injury and a decreasing but persisting post injury for several years [2]. Given the significant risk of morbidity and loss of function with fragility fractures in this population, there is a desire to promote preventative and therapeutic options to optimize bone health [3].

While there is a robust body of literature regarding vitamin D and bone health management after chronic TSCI, evidence and best practices in the acute setting are in comparison sparse [4]. Available studies that examine markers of calcium homeostasis dysfunction after acute traumatic SCI/D, such as when patients are admitted to inpatient rehabilitation facilities (IRFs), have noted vitamin D deficiency develops early after injury following the pattern of post injury bone resorption [2, 5–7]. However, initial hypercalcemia in the post traumatic period precludes the immediate use of Vitamin D supplementation until resolution months later, and in general there is no standard guideline for SCI induced calcium dysregulation management in the acute setting [2, 4].

In addition to IRFs, another site available to review for initial calcium homeostasis dysfunction would be acute care hospitals, which may have data evaluating initial laboratory markers and imaging, and may potentially serve as initial target times for treatment in the future when codified guidelines are created. In particular, data from acute hospitals assessing initial changes in serum and urinary calcium post-acute injury, along with associated lab and imaging values reflective of calcium dysregulation (parathyroid hormone [PTH], Vitamin D in various forms, phosphorus, N-terminal telopeptide, alkaline phosphatase, dual energy x-ray absorptiometry [DXA]) may help inform acute pathophysiology as well as ultimately developing standardized guidelines for treating acute calcium homeostasis dysfunction in traumatic SCI/D patients, particularly since there is no current consensus on treatment [2].

Calcium homeostasis dysregulation is associated with sequelae after acute traumatic SCI/D, and based on available literature this pathophysiology is poorly examined in the acute care setting [4]. This present study hopes to address this gap by examining whether calcium homeostasis markers were checked for patients admitted to an acute level 1 trauma center after initial traumatic SCI during initial hospitalization. It was hypothesized that besides calcium, most commonly used calcium homeostasis markers would not have been regularly checked. The findings of this present study would add to the sparse literature on how calcium dysregulation is evaluated after acute traumatic SCI/D and ideally inform future discussions on creating evaluation protocols.

## Methods

### Subjects

Institutional Review Board (IRB) exempted approval was obtained to retrospectively review patient charts to determine the frequency and values of calcium homeostasis markers in patients who sustained an acute traumatic SCI/D who were initially hospitalized to the institutional level 1 trauma center.

#### Inclusion criteria

Patients ages 18–89 with an acute traumatic SCI/D treated at the local institution with available and accessible chart data.

#### Exclusion criteria

Patients were excluded if they sustained an acute non-traumatic spinal cord injury (NTSCI), had a prior history of any type of SCI/D, or had a premorbid history of a condition causing calcium derangements (i.e., premorbid causing osteopenia, osteomalacia, osteoporosis, hypo or hypercalcemia, hypo or hyperphosphatemia, hypo or hyperparathyroidism, a malignancy with bony involvement, or a condition causing osteonecrosis, sclerosis, or hyperostosis of bone) mentioned on initial admission documentation on manual review.

A combination of ICD codes was provided (Table 1, bolded) to the hospital research informatics team to use as part of the inclusion criteria to define those with SCI/D. This was based on literature review of optimal positive predictive values and specificity of ICD 10 codes for traumatic SCI/D, predominately based on the work of Hagen’s team [8].

**Table 1:** ICD codes related to TSCI.

*ICD-8*	
806.x	Fracture and fracture dislocation of vertebral column with spinal cord lesion
958.x	Spinal cord lesion without evidence of spinal bone injury
*ICD-9*	
344	Other paralytic syndromes
344.0	Quadriplegia and quadriparesis
344.1	Paraplegia
806.x	Fracture of vertebral column with spinal cord lesion
907.2	Late effect of spinal cord injury
952.x	Spinal cord lesion without evidence of spinal bone injury
*ICD-10*	
G82	Paraplegia and tetraplegia
S12.0	Fracture of first cervical vertebra
S12.2	Fracture of other specified cervical vertebra
S13.0	Traumatic rupture of cervical intervertebral disk
S13.2	Dislocation of other and unspecified parts of neck
S13.4	Sprain and strain of cervical spine
**S14.0**	**Concussion and edema of cervical spinal cord**
**S14.1**	**Other and unspecified injuries of cervical spinal cord**
S22.0	Fracture of thoracic vertebra
S23.1	Dislocation of thoracic vertebra
**S24.0**	**Concussion and edema of thoracic spinal cord**
**S24.1**	**Other and unspecified injuries of thoracic spinal cord**
S32.0	Fracture of lumbar vertebra
S33.1	Dislocation of lumbar vertebra
S34.0	Concussion and edema of lumbar spinal cord
**S34.1**	**Other injury of lumbar spinal cord**
**S34.3**	**Injury of cauda equina**
T06.0	Injuries of brain and cranial nerves with injuries of nerves and spinal cord at neck level
T06.1	Injuries of nerves and spinal cord involving other multiple body regions
T09.3	Injury of spinal cord, level unspecified
T91.1	Sequelae of injuries, of poisoning and of other consequences of external causes—Sequelae of injuries of neck and trunk—Sequelae of fracture of spine
**T91.3**	**Sequelae of injuries, of poisoning and of other consequences of external causes—Sequelae of injuries of neck and trunk—Sequelae of injury of spinal cord**

Included codes are bolded. Adapted from Table 1, Hagen et al. [8].

**Fig. 1:**
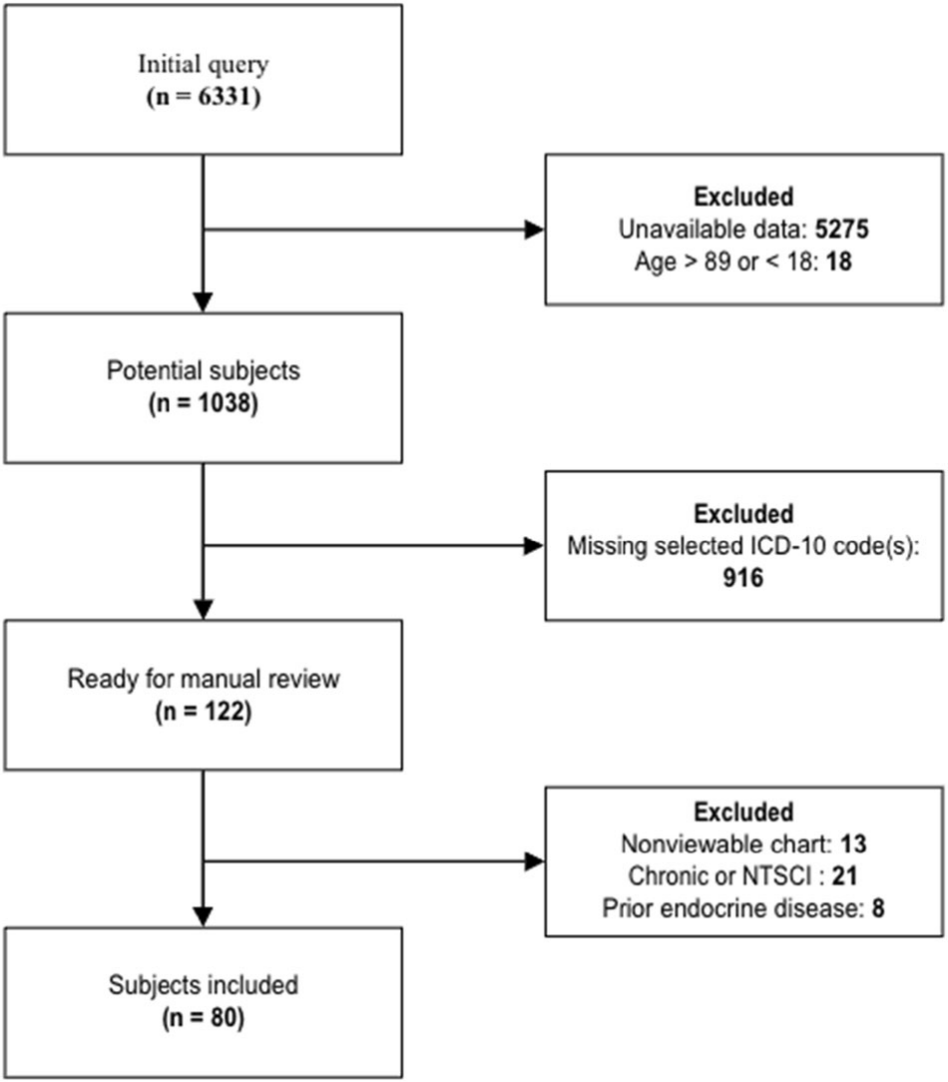
Selection of subjects. Initial query yield 6331 potential subjects; after exclusion criteria applied, 80 eligible subjected remained and were included in the study.

With assistance from our informatics team, patient charts with above criteria were reviewed to see if any of the labs/imaging reports noted in Table 2 were present during the encounter. The data was subsequently reviewed manually by the research team and further filtered to ensure compliance with inclusion/exclusion criteria; of 6331 patients initially queried, 80 ultimately were deemed appropriate for inclusion in the study (Fig. 1).

**Table 2:** Diagnostic tests of interest.

Basic metabolic panel	Vitamin D level (25-hydroxyvitamin D)
Magnesium	C-telopeptide (NTX peptide)
Spot urine calcium	Parathyroid hormone
Testosterone panel	Celiac panel
Liver function tests	DEXA/DXA imaging REPORT

### Measures

#### Outcome measures

The primary outcome measure was the frequency for which specified lab and imaging values of interest were obtained during acute hospitalization for eligible patients.

#### Statistical analysis

Data analysis for descriptive statistics was conducted in IBM SPSS version 29. Summary statistics measured included frequency, mean, and standard deviation.

## Results

**Fig. 2:**
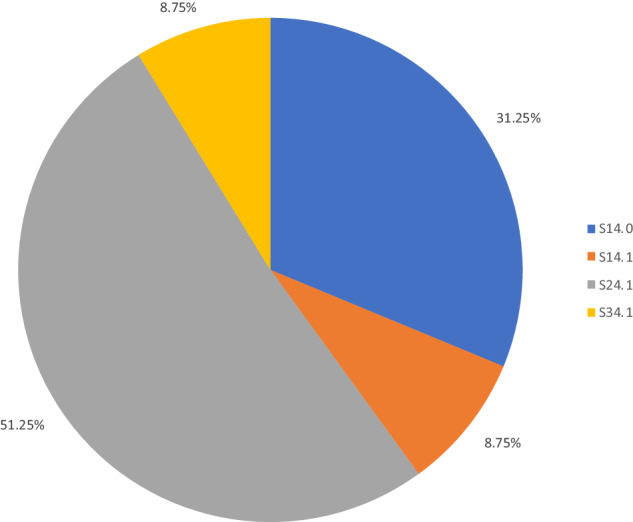
ICD codes of interest present in cohort. The ICD codes starting with S24.1 (representing unspecified injury to the thoracic spinal cord) were the most common diagnostic codes present in this cohort, with the remainder present as smaller percentages of the overall sample.

Summary demographic information for the 80 subjects is listed in Table 3. Note etiology “Other” included other violent means of traumatic SCI besides gunshot wounds, sports injuries, and non-fall workplace related injuries. “Fall” includes both ground level and from height falls. ICD 10 codes reflect the combination previously described by Hagen et al.; note, patients may have had multiple ideal ICD 10 codes overlapping, or even other ICD 10 codes used to describe SCI/D which were not removed [8]. The total frequency of each of the described ICD 10 codes of interest is presented in Table 4 and Fig. 2.

**Table 3:** Demographics.

	n (%)
Sex	
Male	60 (75)
Female	20 (25)
Race	
Black	37 (46)
White	37 (46)
Unknown	3 (4)
Asian	1 (1)
Multiracial	1 (1)
Other	1 (1)
Etiology	
Fall	35 (44)
Motor vehicle accident	25 (31)
Gunshot wound	13 (16)
Other	7 (9)
Age	
18–64	56 (70)
65–89	24 (30)

**Table 4:** Frequency of ICD 10 codes of interest.

ICD 10 code of interest	n (%)
S14.0	25 (31)
S14.1	7 (9)
S24.0	0 (0)
S24.1	41 (51)
S34.1	7 (9)
S34.3	0 (0)
T91.3	0 (0)

**Fig. 3:**
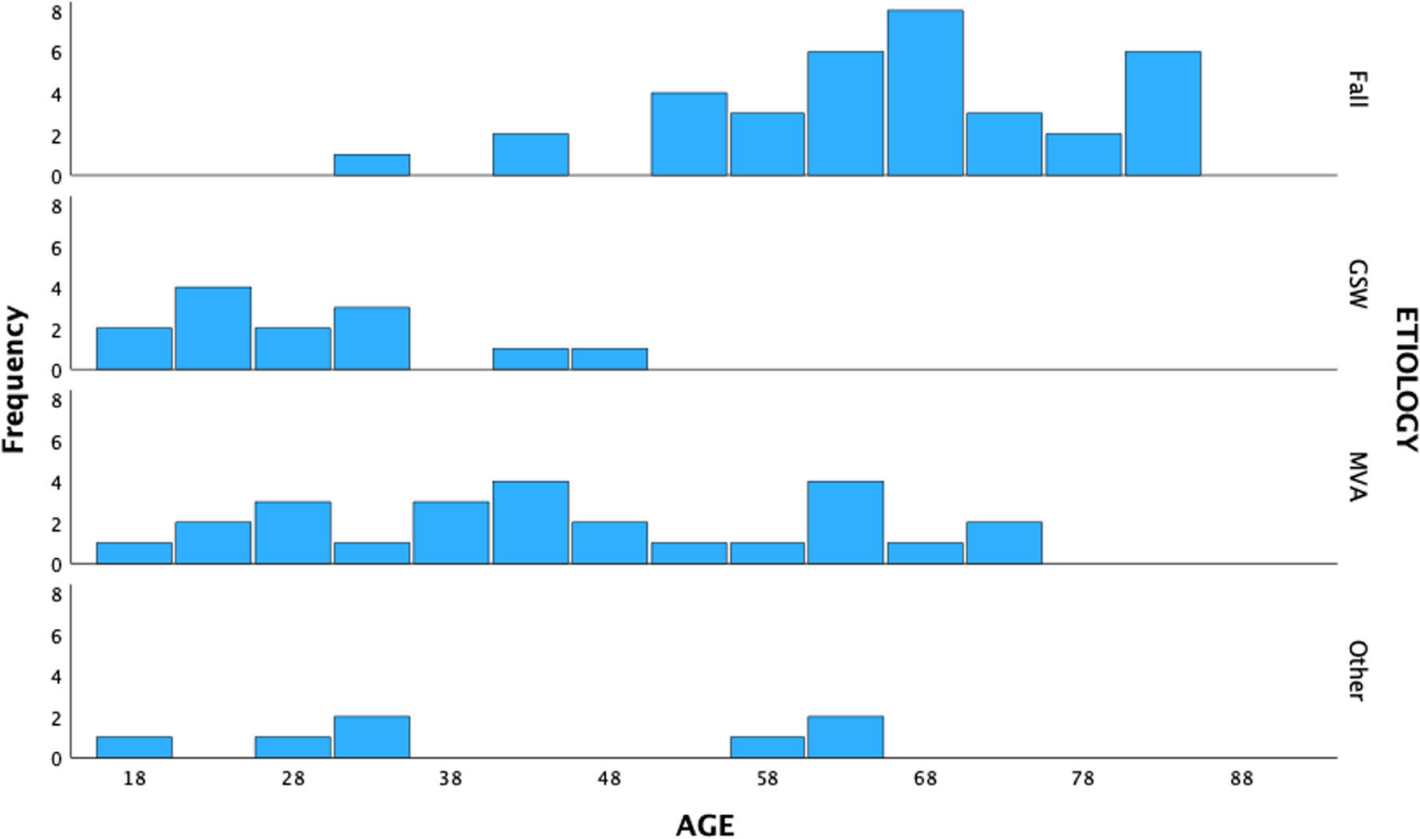
Etiology of injury based on age. Fall, gunshot wounds (GSW), motor vehicle accident (MVA), and Other (aggregated remaining etiologies) and their frequencies in the cohort are displayed.

The average age was 51.3 (SD: 19.2). As described previously in literature [9], there was a general trend for falls as the etiology of injury in older subjects, gunshot wounds (GSW) in the younger population, with a larger spread for motor vehicle accidents (MVA) and variability in the “other” class (Fig. 3).

Of the labs and imaging reports of interest (Table 2), most were not present in this acute population. Of the labs present, values for the lab (or its key component of a panel) average and error/deviation are listed in Table 5. Calcium levels were present in all subjects, likely due to being present in the basic chemistry panel for the institution. Phosphorus was present for nearly every subject. Albumin and alkaline phosphatase were present in less than half of patients, and PTH was only obtained in one subject. Vitamin D was obtained in 12.5% of patients (mean value 28, standard deviation [SD] 23; mean days to check 1.5, SD 1.6).

**Table 5:** Components of available labs of interest.

Lab (serum values)	n (%)	Mean, SD	Mean day to first check, SD
Albumin	34 (42.5)	3.0, 0.59	4.9, 5.7
Calcium (uncorrected)	80 (100)	8.7, 0.99	0.0, 0.85
Vitamin D (all types)	10 (12.5)	28, 23	1.5, 1.6
Phosphorus	76 (95)	3.4, 0.94	0.75, 1.2
Alkaline Phosphatase	33 (41.3)	83, 74	4.6, 5.7
Parathyroid Hormone	1 (1.3)	64, n/a	0, n/a

## Discussion

### Significance of data

As hypothesized, a majority of the labs or imaging reports of interest were not checked in this cohort. This highlights a major roadblock in identifying methods to mitigate the sequelae of calcium dysregulation: failing to identify early trends and points of potential intervention. For example, only 10 of 80, or 12.5% of patients in this cohort had a vitamin D level, in any form, checked during their acute admission. The mean vitamin D level of 28 is comparable to Waliullah et al.’s study of 85 patients with acute TSCI in a trauma center with a mean of 20.5 obtained during the first week from injury, and is typically considered “low” (i.e., below the recommended 40 ng/ml by the Endocrine Society) [4, 10].The results of this study support the need for developing acute care calcium homeostasis lab and imaging protocols to inform development of potential future treatment options – as well as to monitor for sequelae.

Specifically, obtaining and evaluating this information is critical in continuing to explore unique factors that may predispose certain individuals with TSCI to more sequelae than others. For example, Flueck et al. and Oleson et al. noted that certain groups (such as African Americans) have historically been noted to have lower Vitamin D levels, even in able bodied populations, and continue to demonstrate this trend post injury; seasonality (i.e. winter compared to summer) has been noted to adversely affect Vitamin D level as well including in those with SCI/D [1, 4, 11]. Other observed factors that may be associated include age, creatinine, body mass, and sodium levels [7, 12, 13]. Additionally, the role of vitamin D outside of simply bone health is being explored, such as an inflammatory marker and a neuroprotective agent, emphasizing the importance of its evaluation more broadly [13, 14].

At our institution, there is not a standard SCI calcium homeostasis lab order set or protocol. One could be reasonably incorporated at our local institution or at institutions across the nation; while the average acute care length of stay has decreased over time, the most recent estimates of an average of 19 days allows ample time to obtain and interpret calcium homeostasis labs [4, 9]. Future studies incorporating such a protocol could then shift to examining possible medical interventions to ultimately mitigate sequelae of calcium dysregulation.

### Limitations

There were several limitations with this current study. One, a large portion of the potential cohort was removed due to absent patient data from initial queries by the informatics team. However, these were almost completely for injuries more than 3 years prior to the initial start of the study, without any apparent predilection for a patient characteristic. Notably, a recent migration between different electronic health record systems may have led to the loss of data that could be readily queried older than 3 years. Certainly, a larger potential sample size would have been preferred.

Another limitation was related to the classification of SCI/D. The aim was to utilize a method with high specificity to obtain an initial cohort with a high number of actual subjects with SCI/D. This was based on an ICD 10 combination previously found to have optimal specificity and positive predictive value by Hagen et al., though certainly the utilized combination may have excluded some with SCI/D [5]. It is unclear if this could have led to a significant bias in lab results. Notably, thoracic level injuries (predominately ICD code S14.1) were the most common SCI/D presentation in this cohort. This contrasts, however, with established literature [9] noting cervical level injuries (particularly incomplete tetraplegia) as the most common SCI/D, leading to the question of whether this was due to this unique cohort, a rather limited sample size, or a bias introduced from the combination of ICD 10 codes used. One option would have been to look those admitted to an IRF with a Centers for Medicare and Medicaid (CMS) Rehabilitation Diagnosis of spinal cord injury, and then review those initially seen at the acute care hospital and use the associated ICD codes for these patients. However, the concern was that patients who may have died, not transferred to our IRF, or went home may have been missed. Therefore, the literature was reviewed to observe ICD codes that have historically been used to describe SCI to remain more inclusive, and to reduce the risk of selection bias with this alternative. Future medical ontology research for better defining cohorts based on ICD or other related systems would prove useful in mitigating both this issue and more globally for other cohorts.

From a lab perspective, prior endocrine derangements that may have affected calcium homeostasis were reviewed, and subjects with these conditions were excluded; however, often with TSCI, patients may not be able to provide this history initially. Since only initial admission documentation was used to screen for this, it is possible some cases of this were missed.

Finally, this study cannot be generalized to those younger than 18 years of age or older than 89, nor to those with NTSCI with differences in acuity, etiology, pathophysiology, demographics, and outcomes compared to those with TSCI. As this was a single site study, the findings may reflect practices at this single level 1 trauma center and not necessary that of other institutions.

## Conclusions

The aim for this initial study was to evaluate which, if any, calcium homeostasis markers are obtained in the acute setting for patients with TSCI; it was found that as hypothesized, most markers were either infrequently or never checked. We hope to use these results to facilitate the synthesis of a future prospective study where relevant markers are obtained for patients with TSCI acutely as well as serially in the chronic setting. A prospective study of this nature may aid in the further understanding of disorders that develop in these patients and allow for the creation of a calcium homeostasis protocol for this population. If such a protocol is created, we would then aim to examine the feasibility of an interventional study where medical management of calcium dysregulation is initiated in this population and outcomes tracked with markers from the created protocol. In doing so, we aim to minimize acute and chronic sequelae of calcium dysregulation after TSCI to bolster quality of life that may be adversely affected by sequelae of calcium dysregulation.

## Data Availability

This study used a limited data set and thus data is not readily available to be shared in order to maintain patient privacy. Email for further discussion of what may be available upon request.
